# Thermal Storage and Thermal Conductivity Performance of Multi-Dimensional Carbon-Modified 1-Octadecanol Phase Change Materials

**DOI:** 10.3390/molecules31213838

**Published:** 2026-10-23

**Authors:** Xiuli Wang, Zhangyong Hu, Qingmeng Wang, Zhi Liu, Xiaomin Cheng

**Affiliations:** 1 School of Mechatronics and Intelligent Manufacturing, Huanggang Normal University, Huanggang 438000, China; wangxiuli@whut.edu.cn (X.W.); wangqingmeng@whut.edu.cn (Q.W.); lz3839@126.com (Z.L.); 2 School of Materials Science and Engineering, Wuhan University of Technology, Wuhan 430070, China; chengxm@whut.edu.cn

**Keywords:** carbon nanotubes, graphene, carbon foam, 1-Octadecanol, phase change materials, thermal management

## Abstract

The rapid expansion of the renewable energy industry renders thermal energy storage technology a critical support for high-efficiency energy utilization. As an organic phase change material (PCMs), 1-octadecanol (OD) is plagued by intrinsic limitations of low thermal conductivity and sluggish heat absorption release kinetics, restricting its practical deployment under high-power thermal energy storage operating conditions. In this study, carbon nanotubes (CNTs), graphene (GR) and three-dimensional carbon foam (CF) were utilized to modify OD for fabricating a series of composite phase change thermal storage materials (CPCMs). A suite of characterizations including FTIR, SEM, DSC, and Hot Disk measurements were employed to systematically probe the phase composition, microstructure, phase change temperature, phase change enthalpy, stability, and thermal conductivity of CPCMs. Modification effects exerted by carbon-based fillers with varied structures on OD differ remarkably. Three-dimensional network structured CF can effectively build continuous thermal conduction pathways, while GR sheets contribute to optimized interfacial heat transfer performance. In contrast, CNTs are prone to agglomeration and intertwining, which undermines their thermal conductivity enhancement effect. Nevertheless, all CPCMs retain favorable thermal storage capability and superior cycling stability. This work provides meaningful guidance for the rational selection of carbon-based fillers and performance optimization of low-temperature organic PCMs.

## 1. Introduction

The rapid growth of the renewable energy industry has greatly broadened the application scenarios of thermal energy storage technology, which exhibits enormous development potential in fields such as industrial waste heat recovery, battery thermal management, and energy-saving thermal control for buildings [1,2,3]. Nevertheless, wind and solar energy are characterized by inherent intermittency, fluctuation and instability, resulting in poor spatiotemporal matching between energy supply and demand, and further triggering energy waste and system-operation fluctuations [4,5]. Thermal energy storage technology can efficiently store surplus thermal energy and release heat steadily on demand. It serves as a core technology to address the challenge of renewable energy accommodation and improve the comprehensive efficiency of energy utilization, and also represents a research hotspot and key research priority within the renewable energy field.

Latent heat thermal energy storage technology boasts broad research prospects and application markets, with its core advantage lying in the high energy storage density within the phase change temperature range [6]. Organic phase change materials (PCMs) mainly include paraffins, alkanes, esters, fatty acids and alcohols. They possess merits such as high thermal-storage density, low cost and small supercooling degree, yet their universal drawback of poor thermal conductivity hinders large-scale engineering promotion [7,8,9]. Single PCMs still suffer from prominent deficiencies. Insufficient thermal conductivity restricts heat charging and discharging efficiency, which constitutes a critical bottleneck for the practical deployment of latent heat storage systems. As a typical alcoholic organic PCM, 1-octadecanol (OD) delivers a latent heat of up to 240 J/g. It exhibits favorable chemical stability under moderate and low temperature conditions, non-corrosiveness and excellent compatibility with carbon-based fillers. Additionally, it can be blended with other monohydric alcohols to form eutectic systems whose applicable temperature range covers room temperature to 100 °C [10,11]. Nevertheless, OD inherits the intrinsic disadvantages of organic PCMs: low thermal conductivity and severe leakage risk upon melting, which greatly degrade its long-term service reliability.

Research in the field of phase change thermal energy storage currently mainly concentrates on developing novel CPCMs and the characterization of their thermophysical properties. Incorporating carbon-based fillers into phase change matrices represents a mainstream strategy for enhancing thermal conductivity [12]. Carbon materials feature low density, outstanding electrical and thermal conductivity, and favorable chemical compatibility, accompanied by diverse morphological architectures, including one-dimensional carbon nanotubes (CNTs), two-dimensional graphene (GR) and its derivatives, and three-dimensional porous carbon foam (CF). Fillers with different dimensionalities exert remarkably disparate modification effects on PCMs [13,14,15]. Li et al. [16] employed CF/CNT hybrid networks as porous supporting substrates to encapsulate 1-octadecanol and successfully fabricated CF/CNT/OC composite photothermal PCMs. The resultant composites delivered a thermal conductivity of 1.31 W/(m·K) and a photothermal conversion efficiency of 82.6%, alongside desirable thermal-storage performance. Hamza et al. [17] introduced 5 wt % functionalized multi-walled carbon nanotubes (MWCNT-COOH) into pure 1-tetradecanol. The supercooling degree decreased from 10.6 °C to 7.7 °C, while the thermal conductivity (TC) increased by 56.2%. Du et al. [18] established a novel molecular-dynamics simulation approach to predict the structural, diffusive and thermal behaviors of CNT-based composite PCMs. The simulation results revealed that efficient heat-conduction pathways constructed by CNTs could substantially improve the performance of nanocomposite PCMs, provided strong interfacial bonding between components and reduced contact thermal resistance among adjacent CNTs within macroscopic PCM modules. Cheng et al. [19] fabricated PCM-loaded aerogels by dispersing cellulose nanocrystal stabilized PCM emulsions and GR into chitosan solutions, achieving a high melting enthalpy of 185.9 J/g. Ahangar et al. [20] obtained composite carbon foam (CNiF) by pyrolyzing commercial melamine foam (CMF) coated with phenolic resin–NiCl_2_·6H_2_O ethanol solution at 900 °C. Paraffin was then infiltrated into the CNiF to prepare composite thermal-storage materials, which attained a high photothermal-to-thermal conversion efficiency of 66% under an irradiation intensity of 900 W/m^2^. Yu et al. [21] synthesized composite PCMs via solution impregnation by infilling polyethylene glycol (PEG) into porous graphene foam/CNT hybrid frameworks (GCNTs). Molecular-dynamics simulations demonstrated that PEG molecules formed liquid-like layers adjacent to pore walls and crystallized at pore centers, which accounted for the reduction in melting point and latent heat. While carbonaceous fillers have been widely adopted to boost the thermal conductivity of organic PCMs, systematic comparative investigations on multi-dimensional carbon skeletons remain insufficient. The underlying mechanisms governing how carbon skeletons of different dimensionalities regulate the microstructure, thermal storage capacity, thermal conductivity, leakage resistance and cycling stability of organic PCMs still require further systematic clarification.

In this work, three typical multi-dimensional carbon materials, CNTs, GR and CF, were selected to modify OD PCMs, and a series of CPCMs were fabricated. This work focuses on the comparative analysis of performance enhancement of OD PCMs modified by multi-dimensional carbon materials. A variety of characterization techniques including FTIR, SEM, DSC, and Hot Disk measurements were adopted to systematically investigate how different carbon-based fillers affect key thermophysical properties of the composites, such as phase composition, micromorphology, phase change temperature, phase change enthalpy, leakage resistance capacity, thermal cycling stability and thermal conductivity. The interaction mechanisms between carbon fillers of different dimensionalities and OD PCMs were further revealed. The advantages and limitations of one-, two- and three-dimensional carbon skeletons in terms of latent heat retention, thermal-conductivity enhancement and shape stability were comparatively analyzed. Composite phase change systems with outstanding comprehensive performance were screened out. This study provides experimental foundations for the design and fabrication of medium–low-temperature PCMs and possesses practical significance for advancing thermal-energy-storage technologies and facilitating the utilization of renewable energy sources.

## 2. Results and Discussion

### 2.1. Microstructure

**Figure 1:**
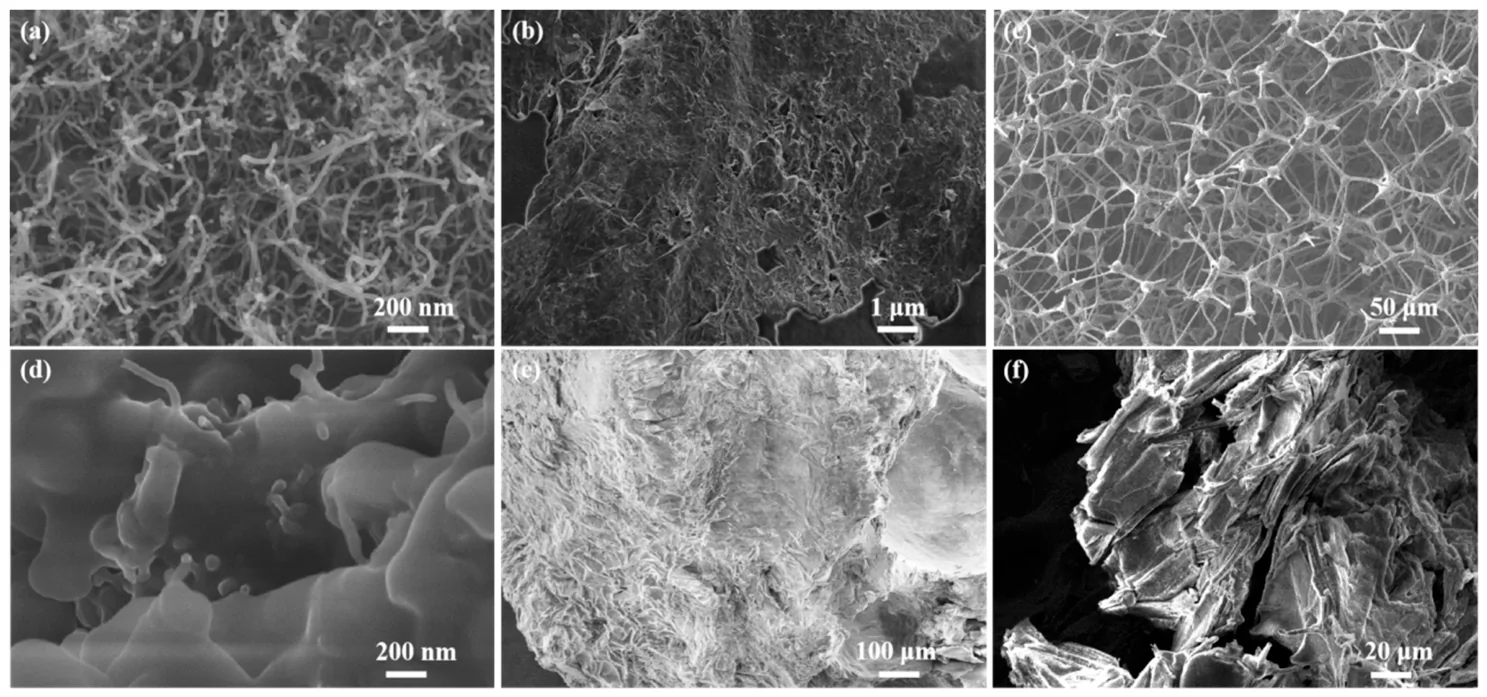
SEM images of (**a**) CNTs, (**b**) GR, (**c**) CF, (**d**) CNTs/OD, (**e**) GR/OD and (**f**) CF/OD CPCMs.

Figure 1 displays SEM images of carbon materials and their corresponding composites. As shown in Figure 1a, CNTs possess an elongated one-dimensional tubular morphology and show a relatively uniform diameter size distribution. The tubes intertwine with one another and form obvious knot-like agglomerations, leaving abundant inter-tube gaps, which makes it difficult to construct continuously interconnected networks. Figure 1b presents the morphology of GR, which shows thin sheets with wrinkled stacking and dense local agglomerates. Only limited local heat-transfer pathways can be established, leading to poor continuity of the overall network. Figure 1c shows three-dimensional CF, which possesses an interconnected three-dimensional porous skeleton with evenly distributed pores. The skeleton remains intact and continuous without filler agglomeration. Figure 1d corresponds to the CNTs/OD CPCMs. OD coats the surface of CNTs, whereas CNTs still suffer from intertwining and agglomeration. Some tubular structures are encapsulated by the PCMs, resulting in poor dispersion homogeneity of fillers and failure to form continuous thermal conduction pathways. Figure 1e shows GR/OD CPCMs. GR nanoflakes attach to the surface of CF skeletons and interpenetrate into pores. The primary porous framework of CF is well preserved, though stacking and agglomeration of GR sheets can be observed in local regions. Figure 1f illustrates the cross-sectional microstructure of CF/OD CPCMs. The pores of CF are filled with OD, while inter-fiber connectivity is maintained. Relying on its porous architecture and capillary force, porous CF can firmly adsorb OD within its pores and effectively suppress leakage during phase transition processes [22,23]. By comparing the micromorphologies of the three carbon-based fillers, it can be found that CNTs tend to intertwine and agglomerate, while GR is prone to sheet stacking. Both of them struggle to form globally continuous thermal conduction networks inside OD. In contrast, three-dimensional CF features interconnected porous skeletons that can accommodate OD, construct continuous thermal conduction channels within composite systems, and provide favorable structural foundations for heat transfer.

### 2.2. Component

**Figure 2:**
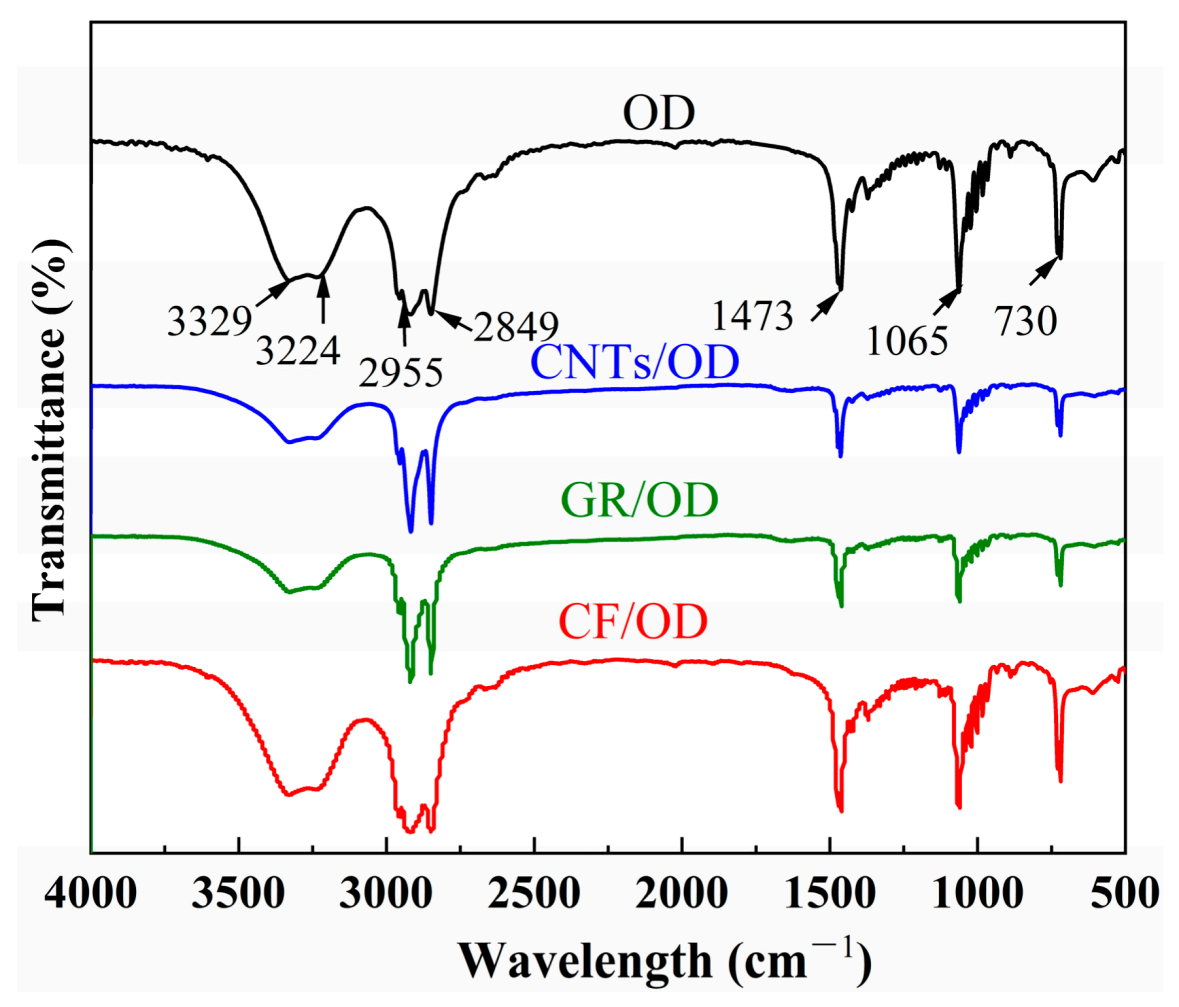
FTIR spectra of OD, CNTs/OD, GR/OD and CF/OD CPCMs.

Fourier transform infrared (FTIR) spectroscopy analysis was utilized to characterize the chemical structure and interfacial bonding mode of each component in the composites, so as to determine whether physical mixing or chemical reaction occurs between carbon-based fillers and OD. FTIR measurements were carried out on pure OD and various CPCMs to investigate whether the incorporation of CNTs, GR and three-dimensional CF alters the molecular structure of OD. Figure 2 presents the FTIR spectra of pure OD, CNTs/OD, GR/OD and CF/OD CPCMs. For pure OD, absorption peaks assigned to -OH stretching vibrations appear at 3329 cm^−1^ and 3224 cm^−1^. The characteristic peaks at 2955 cm^−1^ and 2849 cm^−1^ correspond to saturated C-H stretching vibrations; the peak at 1473 cm^−1^ is attributed to C-H bending vibration; the band at 1065 cm^−1^ belongs to C-O stretching vibration; and the peak at 730 cm^−1^ is ascribed to the rocking vibration of long-chain alkyl groups. All the above-mentioned bands are characteristic functional-group absorption peaks of OD [24]. Comparison of the FTIR spectra of composite samples reveals that the main characteristic absorption peaks of CNTs/OD, GR/OD and CF/OD CPCMs are basically consistent with those of pure OD. No obvious new characteristic peaks are observed, and no remarkable shift of original peaks takes place. The interactions between OD and CNTs, GR as well as three-dimensional CF are merely physical combinations without the formation of new chemical bonds or chemical reactions. The introduction of carbon-based fillers does not destroy the intrinsic molecular structure of OD. The bonding between fillers and PCMs in composite systems originates mainly from physical interactions, and thus the chemical structure of the materials remains stable.

### 2.3. Thermal Storage Properties

**Figure 3:**
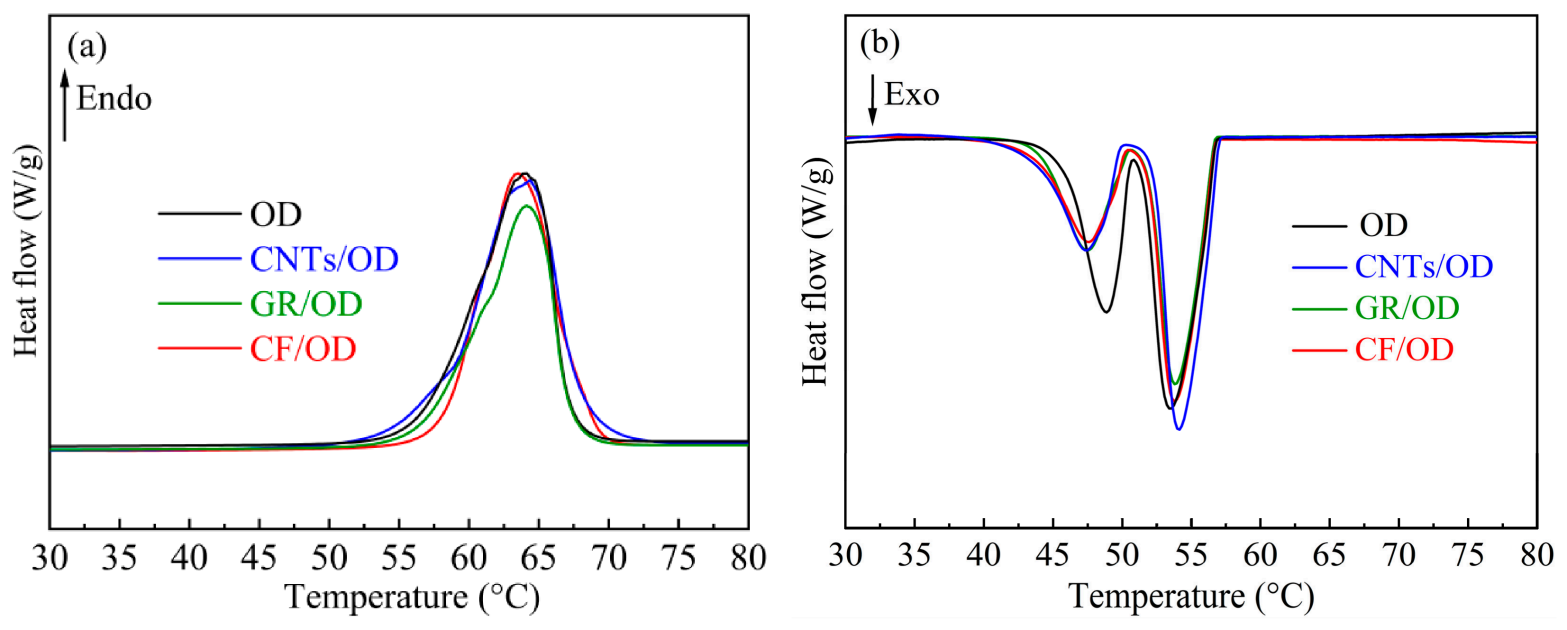
(**a**) DSC melting curves and (**b**) DSC solidification curves of OD, CNTs/OD, GR/OD and CF/OD CPCMs.

Differential scanning calorimetry (DSC) was employed to characterize the thermal properties of pure OD, CNTs/OD, GR/OD and CF/OD CPCMs. The test aimed to investigate the effects of different carbon-based fillers on the phase change temperature, phase change enthalpy and melting–solidification thermal behavior of OD, and evaluate the thermal storage characteristics of the CPCMs. Table 1 presents the DSC test data of pure OD CNTs/OD, GR/OD and CF/OD CPCMs. According to the DSC curves and phase change parameters shown in Figure 3, pure OD exhibits a melting temperature of 57.7 °C and a melting enthalpy of 242.2 J/g, while its solidification temperature and solidification enthalpy are 56.7 °C and 210.1 J/g, respectively. The melting temperatures of CNTs/OD, GR/OD and CF/OD slightly decrease to 57.1 °C, 57.0 °C and 56.9 °C, and their solidification temperatures rise marginally. Carbon fillers can act as heterogeneous nucleation sites, reducing the supercooling degree of the system to a certain extent. The phase change temperatures of all samples are concentrated within the range of 56.9–57.1 °C, demonstrating that the doping of carbon fillers does not significantly alter the operating phase change temperature range of OD PCMs.

Since CNTs, GR and CF themselves have no solid–liquid thermal energy storage capacity via phase transition, the mass dilution effect of fillers leads to lower phase change enthalpy for all composite samples compared with pure OD. Among them, CNTs/OD and CF/OD possess melting enthalpies of 238.8 J/g and 239.0 J/g, showing slight enthalpy attenuation. In contrast, the melting enthalpy of GR/OD decreases to 234.2 J/g, the lowest value among the three composites. Stacking of GR sheets restricts the ordered crystallization of OD molecules and reduces the fraction of effective crystalline components. The interfacial interaction between carbon materials and OD disturbs the arrangement of long-chain alkyl molecules. On one hand, carbon materials provide abundant heterogeneous nucleation interfaces to lower the nucleation barrier for crystallization, facilitate OD crystallization and mitigate supercooling [25,26]. On the other hand, the spatial confinement effect of carbon fillers restricts the regular arrangement of OD molecular chains. When fillers agglomerate and stack, the confinement effect intensifies, inhibiting crystal growth and reducing crystallinity, which further results in a remarkable drop in phase change enthalpy.

The DSC curves reveal that all samples display a single sharp endothermic peak during melting, and the solidification process presents the typical bimodal exothermic feature of long-chain alkanols. This indicates that the incorporation of carbon fillers does not change the phase transition and crystallization mechanism of OD but merely modulates crystallization kinetics. Comprehensive comparison shows that CNTs/OD and CF/OD have advantages in retaining high thermal storage capacity. They can achieve thermal conductivity enhancement at the cost of a slight loss of latent heat, whereas GR modification causes an obvious reduction in thermal storage capacity. In summary, carbon-based fillers regulate the crystallization behavior of OD through the dual effects of heterogeneous nucleation and spatial confinement. They slightly affect phase change temperature and thermal storage enthalpy without changing the intrinsic nature of the phase transition.

**Figure 4:**
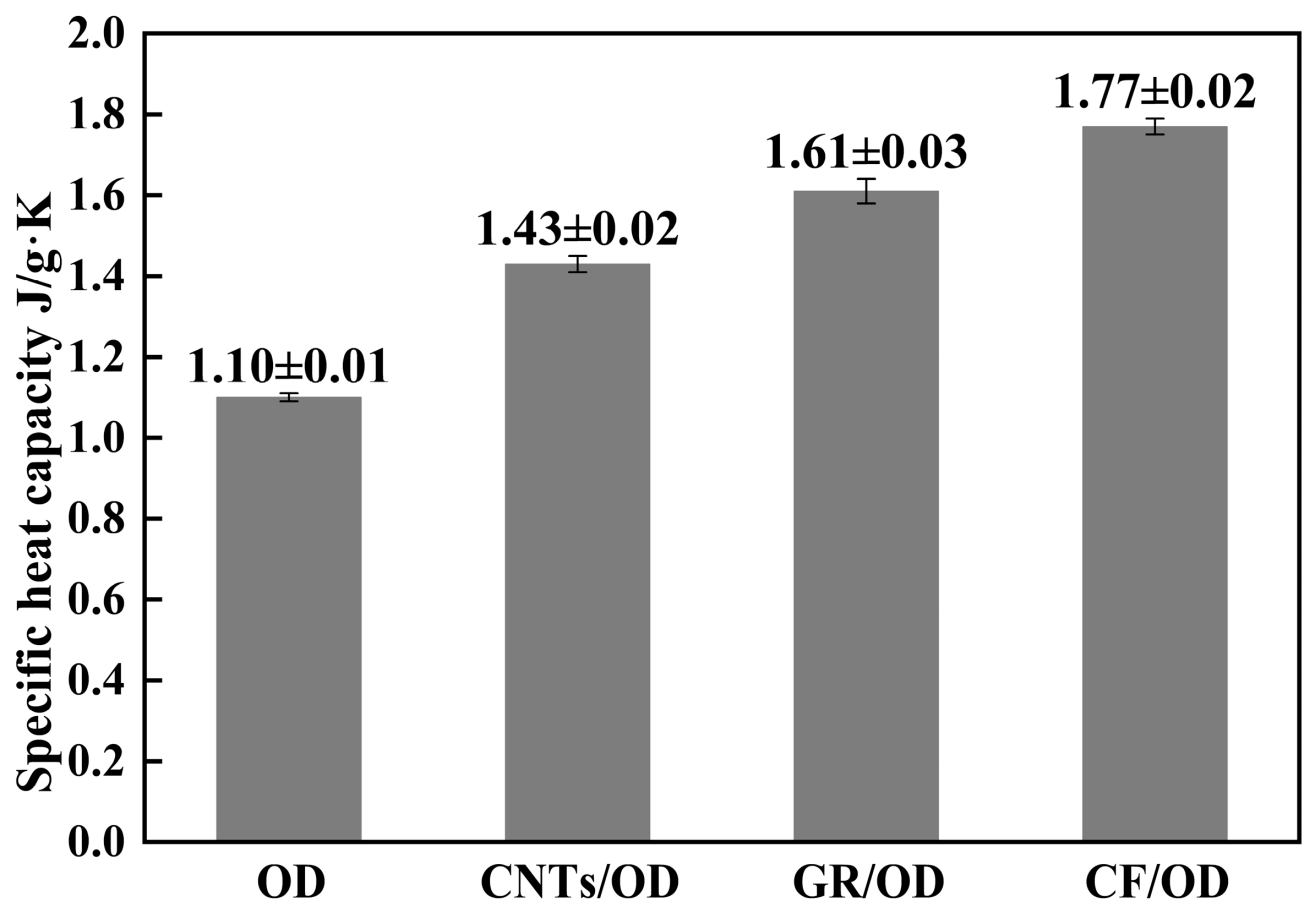
Specific heat capacity of OD, CNTs/OD, GR/OD and CF/OD CPCMs.

Specific heat capacity represents the heat required to raise the temperature of a unit mass of material by one unit and serves as a core parameter for evaluating sensible heat storage capacity. A higher specific heat capacity indicates that the material can store more heat in the non-phase-change temperature range [27]. Figure 4 presents the measured specific heat capacity of OD, CNTs/OD, GR/OD and CF/OD CPCMs. Pure OD exhibits a specific heat capacity of 1.10 J/g·K. Incorporation of carbon-based fillers leads to a remarkable rise in the specific heat capacity for all CPCMs; the values of CNTs/OD, GR/OD and CF/OD reach 1.43 J/g·K, 1.61 J/g·K and 1.77 J/g·K, respectively. The incorporation of carbon skeletons constructs continuous thermal conduction networks. Meanwhile, the interfacial interaction between carbon fillers and OD alters the motion characteristics of molecular chains, which jointly elevates the specific heat capacity of the system [28,29]. Among all samples, CF/OD achieves the optimal specific heat capacity, which is attributed to the porous and continuous structure of three-dimensional CF. Compared with one-dimensional CNTs and two-dimensional GR, CF enables more sufficient interfacial contact and thermal transport pathways. Apart from latent heat of phase change, it further boosts the overall heat storage capacity of the material, which helps broaden the application potential of CPCMs in thermal energy storage and temperature regulation.

### 2.4. Heat Transfer Performance Analysis

**Figure 5:**
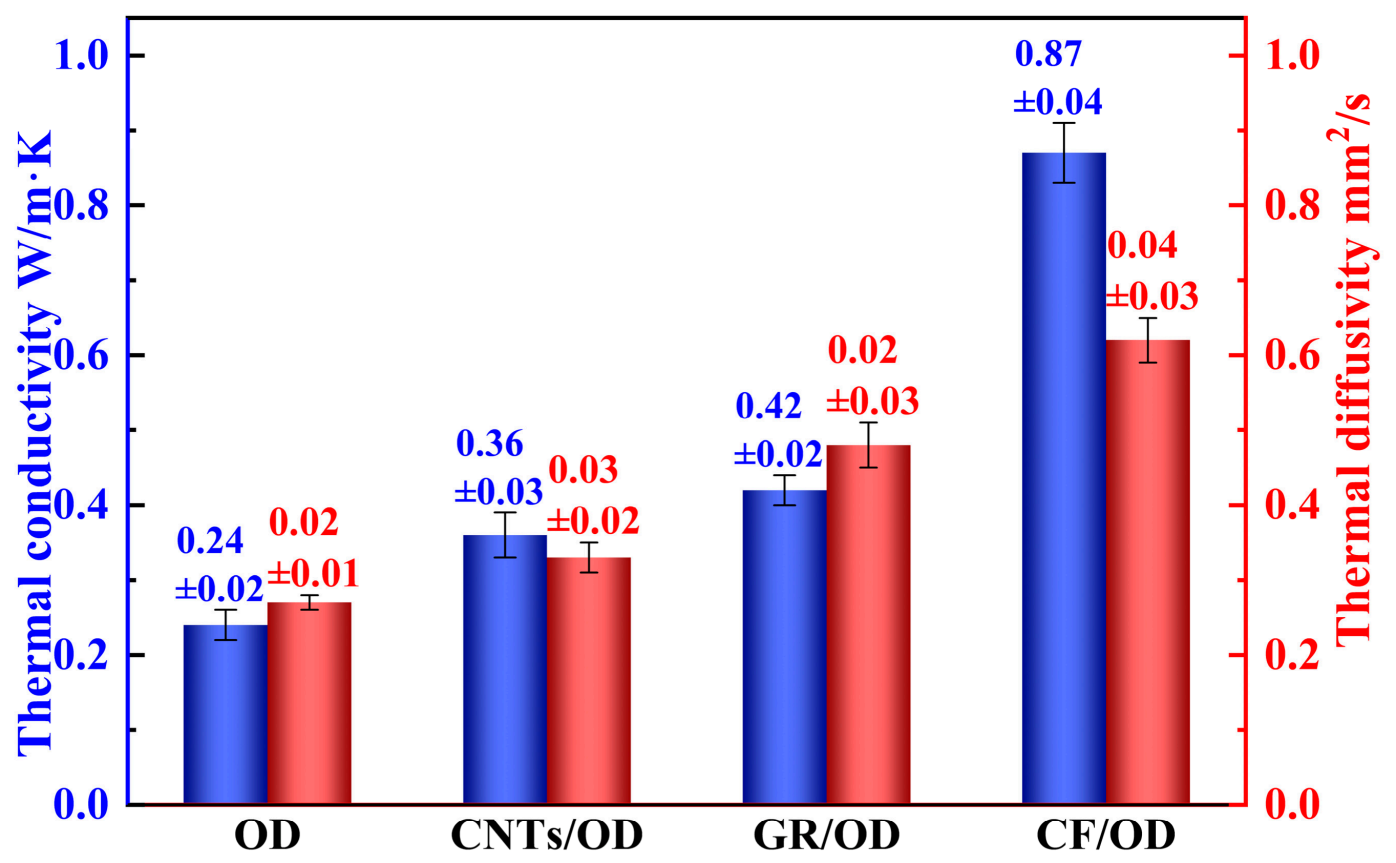
Thermal conductivity and thermal diffusivity of OD, CNTs/OD, GR/OD and CF/OD CPCMs.

A thermal conductivity tester was used to measure the thermal conductivity and thermal diffusivity of pure OD, CNTs/OD, GR/OD and CF/OD CPCMs, so as to explore the enhancement effect of different carbon-based fillers on the heat transfer properties of OD. As shown in Figure 5, pure OD possesses a low thermal conductivity of only 0.24 W/(m·K) and a thermal diffusivity of 0.27 mm^2^/s, reflecting the common drawback of poor heat conduction capacity for organic PCMs. The CNT/OD sample shows a thermal conductivity of 0.36 W/(m·K) and a thermal diffusivity of 0.33 mm^2^/s; GR/OD exhibits a thermal conductivity of 0.42 W/(m·K) and a thermal diffusivity of 0.48 mm^2^/s. CF/OD delivers the most prominent improvement, with thermal conductivity reaching 0.87 W/(m·K) and a thermal diffusivity of 0.62 mm^2^/s. Relative to pure OD, the thermal conductivities of CNTs/OD, GR/OD and CF/OD CPCMs achieve increments of 50%, 75% and 263%. Carbon materials inherently feature ultrahigh intrinsic thermal conductivity. For CF, carbon fibers interconnect within OD, which facilitates the construction of a continuously penetrating three-dimensional carbon thermal conduction network. This shortens the phonon transport path and greatly reduces phonon scattering loss at the interfaces of the organic PCMs. In contrast, CNTs are prone to agglomeration, and stacking of two-dimensional GR sheets increases interfacial thermal resistance. It is difficult for both of them to form long-range continuous three-dimensional heat transfer architectures, resulting in weaker thermal conductivity enhancement compared with the CF system [30,31,32]. The comparison demonstrates that CF achieves efficient heat transfer relying on its three-dimensionally interconnected carbon skeleton and presents the best thermal reinforcement performance. GR and CNTs can only build heat transfer pathways in local regions, and their thermal conductivity improvement is limited by filler dispersion state and interfacial thermal resistance. In summary, carbon-based fillers can construct thermal conduction pathways inside OD, and the 3D continuous carbon skeleton formed by CF serves as the core reason for its remarkable thermal conductivity enhancement.

### 2.5. Resistance to Leakage

**Figure 6:**
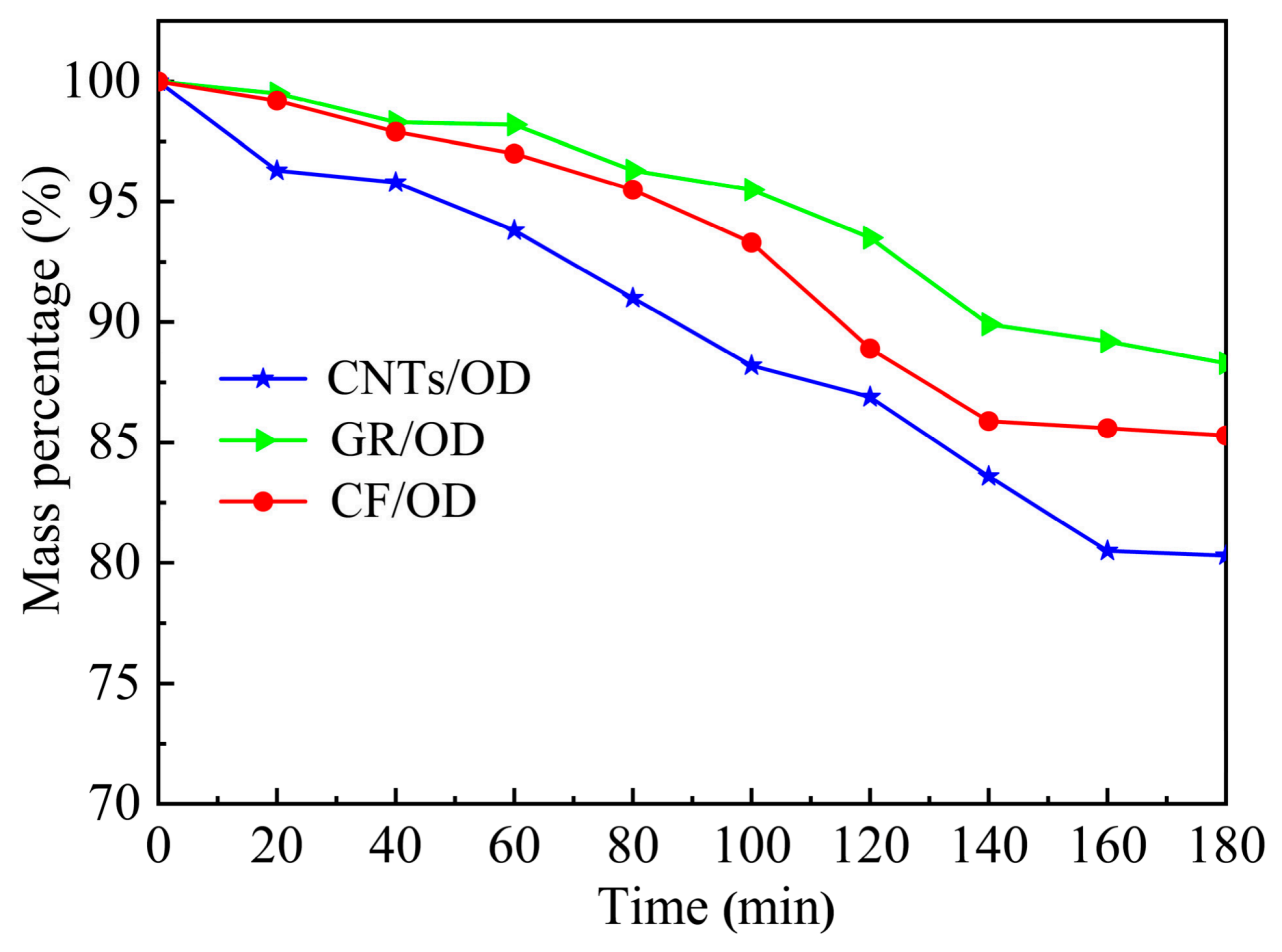
Leakage prevention test of CNTs/OD, GR/OD and CF/OD CPCMs.

Leakage tests were carried out to evaluate the shape stability and leakage resistance of CPCMs. Approximately 300 ± 10 mg of pure OD, CNTs/OD, GR/OD and CF/OD CPCMs were placed in a mold and pressed into circular pellets with a diameter of 30 mm. The pellets were then transferred into Petri dishes lined with three layers of filter paper, and each sample was covered with another three layers of filter paper. The Petri dishes containing samples were placed in a drying oven at 100 °C (about 40 °C above the melting point of OD) for 3 h. The samples were taken out and weighed every 20 min to calculate the mass loss rate. Figure 6 shows the leakage ratio of the CPCMs. Without encapsulation, pure OD completely melted within 20 min. As the test proceeded, the leakage ratios of CNTs/OD, GR/OD and CF/OD continuously increased, indicating different degrees of leakage of OD. After the 180 min isothermal leakage test, the final mass retention rate of CNTs/OD was 80.5%, CF/OD was 85.6%, and GR/OD reached 89.2%. The comparison reveals that GR/OD possesses the best leakage resistance. The two-dimensional sheet-like structure can form a dense barrier to effectively restrain the migration and exudation of OD molecules. CF/OD ranks second; the porous skeleton of three-dimensional CF provides physical confinement for the PCMs. CNTs/OD exhibits the most severe leakage, since one-dimensional CNTs can hardly form a continuous and dense barrier network and only deliver limited confinement effect on OD PCMs. The results demonstrate that the micromorphology of carbon fillers exerts a remarkable influence on the anti-leakage performance of CPCMs. The sheet barrier effect of GR is more beneficial to improving the structural stability of composites and reducing the leakage risk of OD during phase transition, which provides important support for the practical engineering deployment of CPCMs.

**Figure 7:**
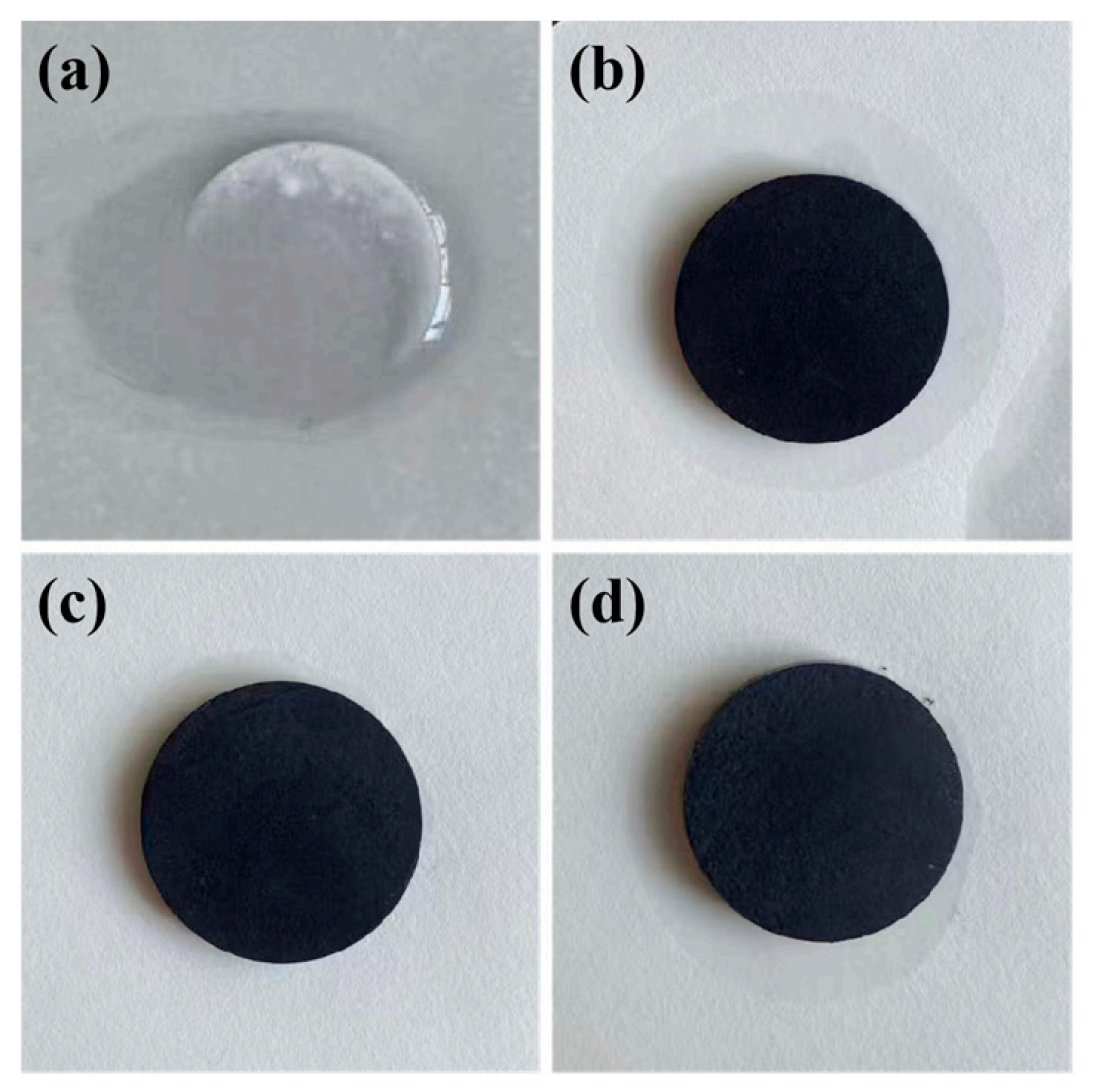
Photographs of (**a**) OD, (**b**) CNTs/OD, (**c**) GR/OD and (**d**) CF/OD CPCMs after thermal treatment at 100 °C for 20 min.

Figure 7 presents digital photographs of the samples captured at 20 min during the leakage test, enabling direct visual comparison of the confinement effect exerted by different carbon-based fillers on OD. In Figure 7a, severe leakage occurs for the pure OD sample; liquid OD forms a large wet area on the filter paper, with substantial outward exudation of OD. Figure 7b shows obvious liquid spreading traces at the edges of the CNTs/OD sample, indicating considerable leakage of PCMs. For the GR/OD sample in Figure 7c, almost no exudation traces are observed on the filter paper, and the sample maintains its shape intact, demonstrating outstanding leakage resistance. Only slight leakage is detected for the CF/OD sample in Figure 7d, and its confinement performance is superior to that of CNTs/OD. These visual observations are consistent with the trend obtained from the mass loss test, confirming that the two-dimensional GR sheet structure can build a barrier layer to effectively suppress the migration of OD and enhance the structural stability of CPCMs.

### 2.6. Reliability and Reversible Stability

To verify the thermal stability and reliability of the CPCMs, thermal cycling tests were performed for 200 cycles within a temperature range of 30–90–30 °C. Table 2 summarizes the thermal performance of pure OD, CNTs/OD, GR/OD and CF/OD CPCMs after 200 heating–cooling cycles. After repeated thermal cycles, no obvious shifts were observed in the melting and solidification temperatures of all samples, and the phase change temperatures remained within the range of 56–57 °C, demonstrating that the incorporation of carbon-based fillers does not substantially alter the phase change behavior of OD.

Pure OD has a melting latent heat of 239.3 J/g and a solidification latent heat of 208.4 J/g after 200 thermal cycles. The latent heat values of the CPCMs decrease because carbon fillers themselves possess no latent heat of phase transition. After cycling, CNTs/OD exhibits a melting latent heat of 201.5 J/g with a latent heat retention rate of 84.4%; GR/OD shows a melting latent heat of 214.3 J/g with a retention rate of 91.5%; CF/OD delivers a melting latent heat of 214.6 J/g with a retention rate of 89.8%. Compared with CNTs/OD, GR/OD and CF/OD exhibit superior cycling stability with less latent heat attenuation. The considerable latent heat loss of CNTs/OD originates from OD leakage and carbon material interface deterioration during cyclic operation. The two-dimensional GR sheets and 3D CF skeleton exert physical confinement on OD, restraining the migration and loss of OD during phase transition and effectively preserving the thermal storage performance of the CPCMs. Overall, the microstructure of carbon-based fillers imposes a remarkable effect on the thermal cycling durability of CPCMs. GR and CF systems possess better long-term service potential.

The critical parameters for CPCMs are thermal enthalpy and thermal conductivity. As a comparison, the thermal properties of related CPCMs reported in the literature are listed in Table 3 [33,34,35,36,37]. It is notable that various fillers and PCM combinations have been developed to enhance the thermal conductivity and thermal stability of phase change materials. Generally, most CPCMs suffer from a reduction in thermal enthalpy when improving thermal conductivity, which indirectly decreases their practical application efficiency. The graphene oxide/graphene nanoplates/paraffin shows a relatively high thermal conductivity but low latent heat [34], while the TiO_2_/PEG composite delivers a low thermal conductivity of 0.39 W/m·K [35]. By contrast, the CF/OD CPCM fabricated via a simple melt-blending method in this work achieves a high thermal enthalpy of 239.0 J/g and a superior thermal conductivity of 0.87 W/m·K. CF serves as a 3D continuous thermal conductive network, which simultaneously maintains high latent heat and greatly improves heat transfer performance. Moreover, the good thermal reliability and thermal cycle stability confirm that CF/OD is a promising candidate for advanced CPCMs applied in thermal energy storage systems.

## 3. Materials and Methods

### 3.1. Materials

1-Octadecanol (OD, C_18_H_38_O, analytical grade, cat. No. 30137128) and graphene (GR, analytical grade, cat. No. XW011034343980012) were supplied by Sinopharm Chemical Reagent Co., Ltd. (Shanghai, China). Carbon nanotubes were purchased from Beijing Boyu Hi-Tech New Material Technology Co., Ltd. (Beijing, China) (purity > 95 wt.%; tube diameter: 10–20 nm; tube length: 10–30 μm). The carbon foam (CF) was fabricated via high-temperature carbonization of melamine foam in an electric furnace under nitrogen atmosphere. Nitrogen was purged for 20 min to create an inert atmosphere, and the heating rate was controlled at 5 °C/min. The sample was carbonized at 600 °C for 1 h. The as-prepared CF exhibits a density of 5.3 mg/cm^3^, porosity of 99.4%, average pore size in the range of 80–100 μm, and a carbonization yield of 70%. All raw materials were employed as received with no additional purification.

### 3.2. Preparation of CNTs/OD, GR/OD and CF/OD CPCMs

**Figure 8:**
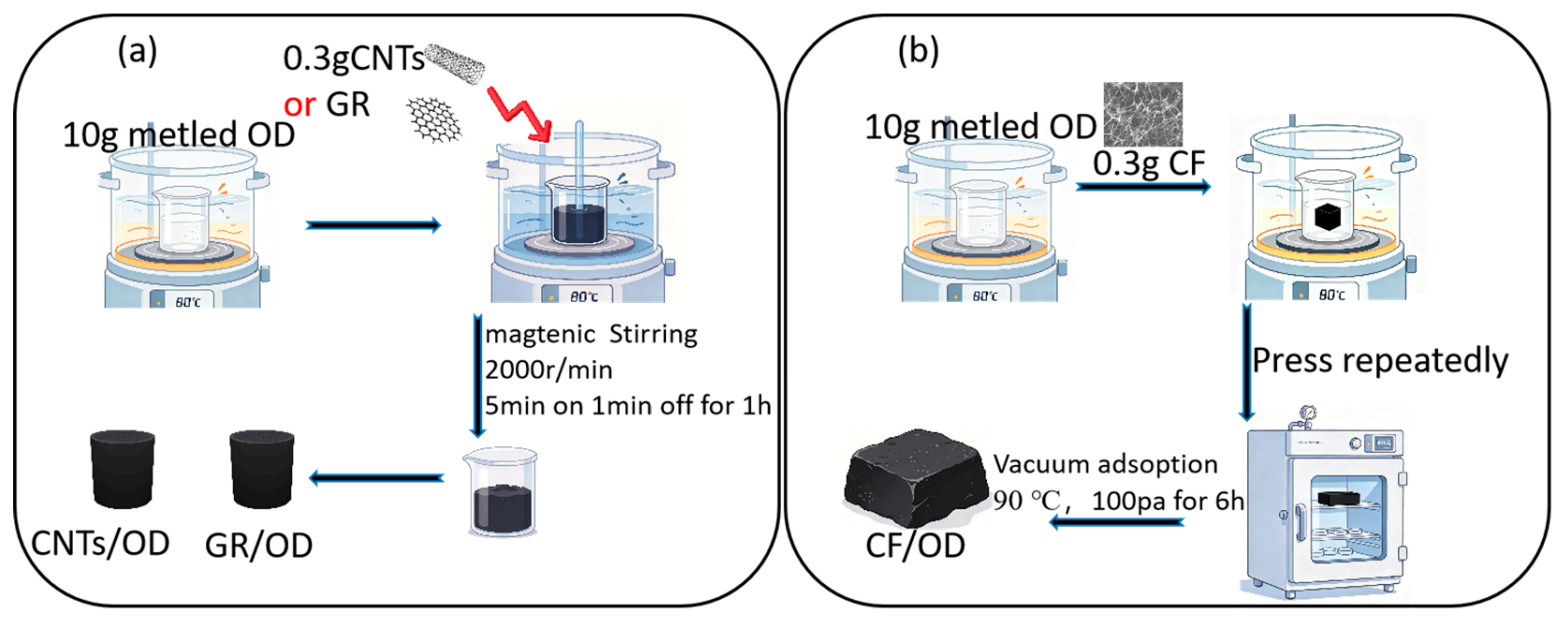
Preparation process of (**a**) CNTs/OD and GR/OD, and (**b**) CF/OD CPCMs.

Figure 8 illustrates the fabrication procedures of CNTs/OD, GR/OD and CF/OD CPCMs. The collecting-type thermostatic heating magnetic stirrer was adjusted to 80 ± 1 °C. Subsequently, 10 g of OD was weighed and transferred into a beaker, which was then placed on the heater for heating. After the OD was fully melted, 0.3 g of CNTs were added slowly. The mixture was first gently stirred with a glass rod to achieve preliminary blending of CNTs and OD. Subsequently, the magnetic stirrer was turned on at 2000 r/min, with an operation cycle of stirring for 5 min followed by a 1 min pause. The total stirring duration was 1 h to obtain a homogeneous mixture. The sample was then cooled and solidified, denoted as CNTs/OD. Pure OD was labeled OD. GR/OD CPCMs were fabricated using the same preparation procedure as CNTs/OD. For the CF/OD sample, 10 g of OD was weighed into a beaker and completely melted in a water bath at 90 °C. Then, 0.3 g of CF was immersed into liquid OD and repeatedly pressed to facilitate sufficient adsorption of molten OD. The sample was transferred into a vacuum drying oven for vacuum adsorption at 90 °C and 100 Pa (absolute pressure) for 6 h. After cooling, the sample was dried and stored, and designated as CF/OD. The detailed mass of CNTs/OD, GR/OD and CF/OD CPCM is shown in Table 4.

### 3.3. Characterization

Field-emission scanning electron microscopy (FE-SEM, JSM-7500F, JEOL, Akishima, Japan) was utilized to examine the surface microstructure and morphological features of the prepared materials. The bulk CPCMs were fractured manually to obtain small fragments. The fractured pieces were mounted onto the sample stage using conductive adhesive. Subsequently, the specimen was sputter-coated with gold prior to FE-SEM observation. A Fourier transform infrared spectrometer (Nicolet 6700) was employed to record the infrared spectra for molecular structure analysis of the materials. FTIR spectra were recorded within 5000–350 cm^−1^ at a resolution of 4 cm^−1^. DSC characterization was performed on a NETZSCH instrument (Selb, Germany). The samples were heated from room temperature to 100 °C at a heating rate of 10 °C min^−1^. A continuous nitrogen stream at 10 mL/min was applied as a protective atmosphere during the experiment. The temperature accuracy is ±0.2 °C. The sample mass loaded into the crucible was 8 ± 0.5 mg. A blank crucible was tested synchronously for baseline correction for each measurement. Each sample was characterized in a single DSC run. A Hot Disk (AB, Gothenburg, Sweden) with a type 7577 sensor and a power of 50 mW was applied to measure the thermal conductivity, thermal diffusivity and specific heat capacity of the CPCMs. The uncertainty analysis of instrumentation is ±2%. Triplicate measurements were conducted on each specimen to secure dependable data, and mean values were taken for subsequent evaluation.

## 4. Conclusions

In this work, CNTs, GR, and three-dimensional CF were adopted as modification fillers to fabricate carbon-based/OD CPCMs. The effects of filler microstructure on the micromorphology, chemical structure, thermal storage capacity, thermal conductivity, leakage resistance, and thermal cycling stability of the composites were systematically investigated.

(1) FTIR results confirm that only physical combination occurs between carbon fillers and OD without chemical reactions or new chemical bond formation, indicating that the chemical structure of OD remains stable after compounding.

(2) The heterogeneous nucleation effect of carbon fillers reduces the supercooling degree of the composite systems and maintains a consistent phase change temperature range with pure OD. Owing to the combined effects of mass dilution and spatial confinement, all CPCMs exhibit a slight attenuation in phase change enthalpy. Among all samples, the CF/OD CPCMs achieve a balanced performance with a high latent heat and a maximum specific heat capacity of 1.77 J/g·K.

(3) The interconnected porous skeleton of three-dimensional CF constructs continuous thermal conduction pathways, enabling CF/OD to deliver a high thermal conductivity of 0.87 W/(m·K), which is significantly superior to that of CNTs/OD and GR/OD. Leakage tests demonstrate that the two-dimensional sheet structure of GR provides an excellent barrier and confinement effect, and GR/OD achieves a mass retention rate of 89.2%. After 200 heating–cooling cycles, both GR/OD and CF/OD exhibit lower latent heat attenuation and superior long-term thermal cycling stability.

## Figures and Tables

**Table 1:** DSC data of OD, CNTs/OD, GR/OD and CF/OD CPCMs.

Samples	Melting		Solidifying	
	T*_m_* (°C)	ΔH*_m_* (J/g)	T*_s_* (°C)	ΔH*_s_* (J/g)
OD	57.7 ± 0.2	242.2	56.7 ± 0.2	210.1
CNTs/OD	57.1 ± 0.2	238.8	56.9 ± 0.2	207.0
GR/OD	57.0 ± 0.2	234.2	56.9 ± 0.2	200.5
CF/OD	56.9 ± 0.2	239.0	56.8 ± 0.2	201.2

**Table 2:** Thermal properties after 200 thermal cycles of OD, CNTs/OD, GR/OD and CF/OD CPCMs.

Samples	Melting			Solidifying		
	T*_m_* (°C)	ΔH*_m_* (J/g)	ω (%)	T*_s_* (°C)	ΔH*_s_* (J/g)	ω (%)
OD (200)	57.2 ± 0.2	239.3	98.8	56.9 ± 0.2	208.4	99.2
CNTs/OD (200)	57.1 ± 0.2	201.5	84.4	56.5 ± 0.2	176.4	85.2
GR/OD (200)	56.8 ± 0.2	214.3	91.5	57.0 ± 0.2	183.1	91.3
CF/OD (200)	56.8 ± 0.2	214.6	89.8	56.1 ± 0.2	181.5	90.2

**Table 3:** Comparison of thermal properties of different CPCMs in the literature.

Filler	Methods	Matrix	T*_m_* °C	Thermal Enthalpy (J/g)	Thermal Conductivity (W/m K)	Ref.
Graphene/moss biomass	Vacuum adsorption	Lauryl alcohol	20.4	161.2	0.43	[33]
Graphene oxide/graphene nanoplates	Microencapsulation	Paraffin	69.1	189.3	0.90	[34]
TiO_2_	Sol–gel	PEG	55.0	123.3	0.39	[35]
CuS/ZnO	Calcination	PA	75.2	155	0.45	[36]
Nano-TiO_2_/carbon nanofiber	Direct addition	OD	57.6	209.3	0.43	[37]
Carbon foam	Melt blending	OD	56.9	239.0	0.87	This work

**Table 4:** The mass of CNTs/OD, GR/OD and CF/OD CPCMs.

Samples	OD/g	Carbon Filler	Carbon Filler/g
CNTs/OD	10	CNTs	0.3
GR/OD	10	GR	0.3
CF/OD	10	CF	0.3

## Data Availability

Data will be made available on request.

## Abbreviations

The following abbreviations are used in this manuscript:

PCMs	Phase change materials
CPCMs	Composite phase change materials
CNTs	Carbon nanotubes
GR	Graphene
CF	Carbon foam
OD	1-Octadecanol
CNTs/OD	Carbon nanotubes/1-Octadecanol
GR/OD	Graphene/1-Octadecanol
CF/OD	Carbon foam/1-Octadecanol
FTIR	Fourier transform infrared spectroscopy
SEM	Scanning electron microscopy
DSC	Differential scanning calorimeter
T*_m_*	Melting temperature
ΔH*_m_*	Melting enthalpies
T*_s_*	Solidification temperature
ΔH*_s_*	Solidification enthalpies

## References

[1] Senobar H., Wahab A., Ahmadi M., Zabihi O., Kemper H., Easton M., Naebe M., Khayyam H. (2026). Phase change materials (PCMs) for thermal energy storage systems. Mater. Today Sustain..

[2] Vallese L., Javadi H., Badenes B., Urchueguia J.F., Lombardo G., Menegazzo D., Ure Z., Cesari S., Bottarelli M., Baccega E. (2026). A comprehensive review of thermal energy storage technologies and their applications: Creation of a database. Renew. Sustain. Energy Rev..

[3] Kumar R., Chauhan P.R., Rathod M.K., Kumar K., Sharma P., Said Z., Choudhary P., Lamba R., Tyagi P.K. (2026). Advancing energy storage systems through machine learning: Current developments, technical challenges, and future directions. Appl. Therm. Eng..

[4] Ma Y., Wang H., Tan H., Li Z., Li X. (2026). Comparative analysis of passive and active heat storage technologies in Chinese solar greenhouse: A framework based on energy efficiency, economic viability, and controllability. J. Energy Storage.

[5] Dhass A.D., Beemkumar N., Sunil Kumar M., Subbiah G., Singh R.P., Priya K.K. (2025). Enhancing solar drying systems through integrated thermal energy storage and solar-assisted heat pump technologies: A pathway to sustainable food processing. Results Eng..

[6] Jatav A.K., Reddy K.S. (2026). Advanced materials and system designs for latent heat storage in solar process heat and power generation. Appl. Therm. Eng..

[7] Togun H., Basem A., Jweeg M.J., Biswas N., Abdulrazzaq T., Shelare S., Soudagar M.E.M., Chattopadhyay A., Paul D., Sadeq A.M. (2026). Advances in inorganic and organic phase change materials for thermal energy storage: A comprehensive review of properties, challenges, and innovations. Appl. Therm. Eng..

[8] Yin G.-Z., Pichel M., Benito E.G., Olazagoitia J.L., Wang D.-Y., Yang X.-M. (2025). Utilizing highly porous mycelium based materials as a sustainable supporter for form-stable organic PCMs. Mater. Today Commun..

[9] Dubey A., Arora A. (2024). Effect of various energy storage phase change materials (PCMs) and nano-enhanced PCMs on the performance of solar stills: A review. J. Energy Storage.

[10] Wang X., Wang Q., Cheng X., Yang Y., Chen X., Cheng Q. (2025). Enhanced Heat Transfer of 1-Octadecanol phase Change Materials Using Carbon Nanotubes. Molecules.

[11] Nguyen G.T., Do M.H., Ly T.N., Park I., Bui T.H. (2022). Novel shape-stabilized phase change materials: Insights into the thermal energy storage of 1-octadecanol/fumed silica composites. J. Energy Storage.

[12] Pandey S., Srivastava A., Mishra H., Kumar A., Sharma A. (2026). Carbon material stabilized fatty acid composite for thermal energy storage. Sustain. Energy Technol. Assess..

[13] Liu Z., Huang X.-H., Wang X.-X. (2026). The role of multi-walled carbon nanotubes in enhancing the thermal performance of phase change materials for sustainable energy storage. Therm. Sci. Eng. Prog..

[14] Zheng W., Xu R., Lin P., Cui W., Mei W., Lin Q., Zhang X. (2026). Three-dimensional porous skeleton-structured carbon foam/stearic acid composite as a high-performance phase change material for thermal energy storage. J. Energy Storage.

[15] Chen L., Li Y., Fei H., Tong J., Dai X. (2025). Properties regulation and application of biomass carbon based composite phase change materials in thermal energy storage. Sol. Energy.

[16] Li S.-Y., Yan T., Huo Y.-J., Pan W.-G. (2024). Carbon nanotube/carbon foam thermal-bridge enhancing solar energy conversion and storage of phase change materials. Mater. Today Sustain..

[17] Ayaz H., Chinnasamy V., Shin M., Cho H. (2023). Investigation of 1-tetradecanol with functionalized multi-walled carbon nanotubes as PCM for high-density thermal energy storage. J. Mater. Res. Technol..

[18] Du Y., Zhou T., Zhao C., Ding Y. (2022). Molecular dynamics simulation on thermal enhancement for carbon nano tubes (CNTs) based phase change materials (PCMs). Int. J. Heat Mass Transf..

[19] Cheng C., Hu L., Liu J., Seidu R.K., Zhang S., Guo R., Xu J., Jiang S.-X. (2026). An eco-friendly method for preparing biomass aerogels with phase change material emulsions and graphene for smart thermal management. Chem. Eng. J..

[20] Ahangar A.M., Rahmani A., Maleki M., Ahmadi R., Razavi S.H. (2024). Highly graphitized carbon foam to construct phase change materials composites for multiple solar−thermal energy conversion. Sol. Energy Mater. Sol. Cells.

[21] Yu Z., Feng Y., Feng D., Zhang X. (2021). Thermal properties of three-dimensional hierarchical porous graphene foam-carbon nanotube hybrid structure composites with phase change materials. Microporous Mesoporous Mater..

[22] Wang C., Feng L., Li W. (2012). Shape-stabilized phase change materials based on polyethylene glycol/porous carbon composite: The influence of the pore structure of the carbon materials. Sol. Energy Mater. Sol. Cells.

[23] Zhao Y., Zheng W., Dong J., Li R., Sheng C., Wen J., Chang C. (2025). The preparation and performance of expanded perlite/carbon-dodecyl alcohol composite thermal storage material. J. Alloys Compd..

[24] Gong S., Cheng X., Li Y., Wang X., Wang Y., Zhong H. (2020). Effect of nano-SiC on thermal properties of expanded graphite/1-octadecanol composite materials for thermal energy storage. Powder Technol..

[25] Wang X., Wang Q., Cheng X., Xiong W., Chen X., Cheng Q. (2024). Thermophysical Investigation of Multiform carbon foam/1-Octadecanol Composite Phase Change Materials for Thermal Management. Molecules.

[26] Mishra A.K., Morciano M., Agegnehu B.W., Campagnoli E., Giaretto V., Bottega A., Fasano M., Mongibello L., Chiavazzo E. (2025). Dynamic PCM strategies with nano-enhanced composites for optimal thermal energy storage and management. Chem. Eng. J. Adv..

[27] Jafari F., Dongellini M., Semprini G., D’Errico G.A., Bonoli A. (2026). Recycled concrete integration with PCM: A sustainable approach to high-efficiency latent heat thermal energy storage systems. J. Energy Storage.

[28] Dimberu G.A., Yun B., Wi S., Kang Y., Kim S. (2021). A comparative analysis of biochar, activated carbon, expanded graphite, and multi-walled carbon nanotubes with respect to PCM loading and energy-storage capacities. Environ. Res..

[29] Hu W., Zeng C., Liu W. (2026). Regulating specific heat with B_4_C in composite PCMs for enhanced thermal buffering of phase change concrete. J. Build. Eng..

[30] Feng C., Liu H., Hang Z., Su Y., Xia X., Weng G.J. (2025). Study on thermal conductivity of 0D/1D/2D carbon filler reinforced cement composites with phonon physical model. Cem. Concr. Compos..

[31] Li Y., Pei Z., Zhu Y., Li M. (2026). High thermal conductivity in superhard carbon nitrides: Phonon transport and thermo-mechanical Co-design. J. Mater..

[32] Yamamoto H., Okano Y., Uchida K., Kageshima M., Kuzumaki T., Miyake S., Takashiri M. (2024). Phonon transports in single-walled carbon nanotube films with different structures determined by tensile tests and thermal conductivity measurements. Carbon Trends.

[33] Sarı A., Can A., Çakır E., Batan N., Kolaylı S., Gencel O. (2025). Graphene/moss biomass/lauryl alcohol composites for thermal energy storage. Mater. Chem. Phys..

[34] Zhou Y., Li C.H., Wu H., Guo S.Y. (2020). Construction of hybrid graphene oxide/graphene nanoplates shell in paraffin microencapsulated phase change materials to improve thermal conductivity for thermal energy storage. Colloids Surf. A.

[35] Cheng X.D., Feng Q.G., Ni W.L., Li X., Qi Y., Zhang S.Y., Wu Q.H., Huang Z.Y. (2024). Stable and reliable PEG/TiO_2_ phase change composite with enhanced thermal conductivity based on a facile sol-gel method without deionized water. J. Energy Storage.

[36] Zhou Z., Huang Y.Q., Shen Q., Li Y.Y., Cheng X.M. (2024). Composite phase change materials with carbon-mesh/CuS/ZnO interface biocarbon skeleton for solar energy storage, solar photocatalysis and electromagnetic shielding. J. Energy Storage.

[37] Qin J., Chen Y., Xu C., Fang G. (2021). Synthesis and thermal properties of 1-octadecanol/nano-TiO_2_/carbon nanofiber composite phase change materials for thermal energy storage. Mater. Chem. Phys..

